# Epidemiological characteristics of amebiasis in Japan from 2001 to 2022

**DOI:** 10.1371/journal.pone.0318901

**Published:** 2026-07-07

**Authors:** Keisuke Iida, Hirotake Mori, Dmytro Remez, Daria Krokva, Yoshiro Hadano, Aongart Mahittikorn, Toshio Naito

**Affiliations:** 1 Department of General Medicine, Juntendo University Faculty of Medicine, Tokyo, Japan; 2 Division of Infection Control and Prevention, Shimane University Hospital, Izumo, Shimane, Japan; 3 Department of Protozoology, Faculty of Tropical Medicine, Mahidol University, Bangkok, Thailand; UNAM FACMED: Universidad Nacional Autonoma de Mexico Facultad de Medicina, MEXICO

## Abstract

Amebiasis cases in Japan are reported to the government according to the Infectious Diseases Control Law. Previous studies have shown significant reductions in total case numbers after 2018 and during the COVID-19 pandemic. This study aimed to clarify the recent trends of amebiasis cases in Japan, including during the pandemic period, with details on places of infection, using government surveillance data from January 1, 2001, to December 31, 2022. Change of time trends were modeled through piecewise mixed-effect regression model with knots set at 2018 and 2020. Year-to-year differences in case numbers were statistically assessed using Poisson regression model. And descriptive analyses of amebiasis cases reported in Japan by sex, age class, and prefecture (from 2001 to 2022) were conducted. Piecewise time trends of male-domestic cases showed increasing trend by 59.5 cases per year (p < 0.0001) before 2008. The trend slowed but still increased during 2008–2018, showing annual increase of 15.2 cases per year (p = 0.0014). A sharp decline occurred during 2018–2020, with cases decreasing by 219.0 cases per year (p < 0.0001). After 2020, the trend did not show statistically significant change (−16.9 cases per year, p = 0.6532). Poisson regression confirmed significant reductions in total and domestic cases between 2017–2018 and 2019–2020, while imported cases declined significantly only between 2019 and 2020. Male cases predominated, with most male cases in their 40s and 50s. Most cases of amebiasis have been reported in metropolitan areas. These results suggest that the decreased case numbers during the COVID-19 pandemic were due to not only the travel ban, but less socioeconomic activity. Furthermore, the epidemiology of amebiasis is similar to that of HIV infection in Japan, but the case numbers of amebiasis have not yet increased through 2022, showing a different trend from HIV infection and syphilis, the reason of which is unclear and needs further investigation.

## Introduction

Amebiasis is an infectious disease caused by *Entamoeba histolytica,* which induces amebic colitis, a major cause of diarrhea in developing countries. In developed countries, though it causes travelers’ diarrhea, men who have sex with men (MSM) are known to have a higher risk of amebiasis. Thus, amebiasis is a sexually transmitted disease (STD), as well as a gastrointestinal infection [1]. It is estimated that amebiasis kills more than 55,000 people each year [2]. In industrialized countries, it is reported that HIV-infected MSM are at higher risks for acquiring invasive amebiasis [3,4].

In Japan, amebiasis is included in notifiable disease surveillance under the Infectious Disease Control Law. For all amebiasis cases in Japan, physicians are required to report relevant information to the government. Therefore, nationwide epidemiological trends in amebiasis cases can be tracked in Japan. Based on this surveillance data [5], several reports have been made so far. Ishikane et al. analyzed amebiasis cases in Japan up to 2013 and reported a steady increase in the number of total cases and in the proportion of domestic cases from 2000 to 2013 [6]. The National Institute of Infectious Diseases (NIID) of Japan published online a partial summary report of the epidemiology of amebiasis for the decade up to 2016 [7]. More recently, Hadano et al. showed that the total number of amebiasis cases reported in Japan reached a plateau after 2014 and identified a significant decrease in the total number of cases after 2018, when commercial distribution of antibody test reagents was stopped [8]. Thereafter, regarding diagnostic testing, serum antibody test reagents were re-approved in 2024. Hirae et al. demonstrated that the total number of amebiasis cases in Japan decreased along with other communicable diseases during the COVID-19 pandemic period [9]. However, to the best of our knowledge, there have not been any reports of the recent epidemiology of amebiasis in Japan, by place of infection (domestic or imported).

The primary objective of this study was to clarify epidemiological trends in amebiasis in Japan in recent years, when there have been changes in diagnostic testing method availability and in the behavior of the general population affected by the COVID-19 pandemic. In addition, summary charts of the epidemiology of amebiasis in Japan up to 2022 (by sex, age class and prefecture), making clear the epidemiological trends in amebiasis in the country, are provided.

## Methods

### Study area and period

This was a retrospective study using data from January 1, 2001, to December 31, 2022, obtained from the National Epidemiological Surveillance of Infectious Diseases (NESID) made available by NIID. This surveillance is conducted throughout in Japan in accordance with the Infectious Diseases Control Law.

### Protocol for reporting amebiasis in Japan

Physicians who diagnose amebiasis are required, under the Infectious Diseases Control Law in Japan, to report each case to the local public health center in 7 days. The report includes information on the patient’s sex and age, disease type (intestinal or extraintestinal), clinical signs and symptoms, diagnostic methods, route of infection, and place of infection. According to the government guideline [10], each amebiasis case is confirmed by one of the following methods: (i) detecting pathogen by microscopy; (ii) detecting antigen of the pathogen by ELISA; (iii) detecting genes of the pathogen by PCR; (iv) detecting antigen of the pathogen by immunochromatography; or (v) detecting antibody. Of the methods listed above, methods (i), (ii), and (iii) use stool, biopsy, or abscess fluid samples, method (iv) uses stool samples, and method (v) uses serum samples. For disease types, the government guideline describes intestinal amebiasis as chronic intestinal infection with symptoms of diarrhea, bloody stool, tenesmus, bloating, and lower abdominal pain or discomfort during defecation. It also describes extraintestinal amebiasis as abscess formation, typically in the liver, mostly caused by spread of the pathogen through the bloodstream. When reporting, physicians select the category into which each case falls. Regarding the place of infection, physicians must classify cases as either domestically acquired or imported. For domestic cases, the place of infection is reported at the prefecture and municipality levels. For imported cases, the country of infection is specified. The place of infection may be reported as either definite or probable.

### Data source

NESID data are tentatively summarized and published weekly. Yearly summaries are provided as annual reports [5], which include tables of notifiable infectious diseases by; week, place of infection, disease type, sex, age-group and prefecture. In the present study, the annual reports through 2022 were further summarized and analyzed. Annual trends in amebiasis cases reported in Japan were summarized by place of infection (domestic or imported, from 2001 to 2022) and by disease type (i.e., intestinal and/or extraintestinal amebiasis, from 2008 to 2022). In addition, summary charts of amebiasis cases reported in Japan by sex, age class and prefecture (from 2001 to 2022) were created using the NESID data.

### Statistical analysis

Male-domestic cases exhibit extremely higher prevalence than other cases (male-imported, female-domestic, and female-imported cases). Therefore, we investigated time trends of male-domestic amebiasis case numbers. Considering the previous reports which reported the effects of cessation of commercial distribution of testing reagents [8] and the COVID-19 pandemic [9] on epidemiological trends of amebiasis in Japan, we specified a priori years 2018 and 2020 as potential time points where trends have changed. To analytically examine time trend of the numbers of reported amebiasis cases in Japan, with focus on changes in 2018 and in 2020, change of time trend at several time periods were modeled through piecewise mixed-effect regression model with first-order autocorrelational structure (AR1). Amebiasis cases were assumed to follow normal distribution in this model (see S1 Appendix Table A). The piece-wise regression model contains three spline functions which reflect the changing rate of time trend. Furthermore, to test hypotheses that the reduction of case numbers between years 2017 and 2018, and between 2019 and 2020 were statistically significant, the number of cases were modelled by Poisson regression, and log ratios of years listed above were tested by Wald test. For all analyses, p values were two-sided, and p < 0.05 was considered significant. Statistical analyses were carried out by SAS 9.4 (SAS Institute Inc., Cary, NC, USA).

### Ethical approval

This study was not subject to ethics application. Permission to use the NESID data is on the NIID’s website [11], and the data is widely used for academic research. The NESID data is already anonymised when it is made publicly available, therefore authors did not have access to information that could identify individual participants during or after data collection.

## Results

**Fig 1:**
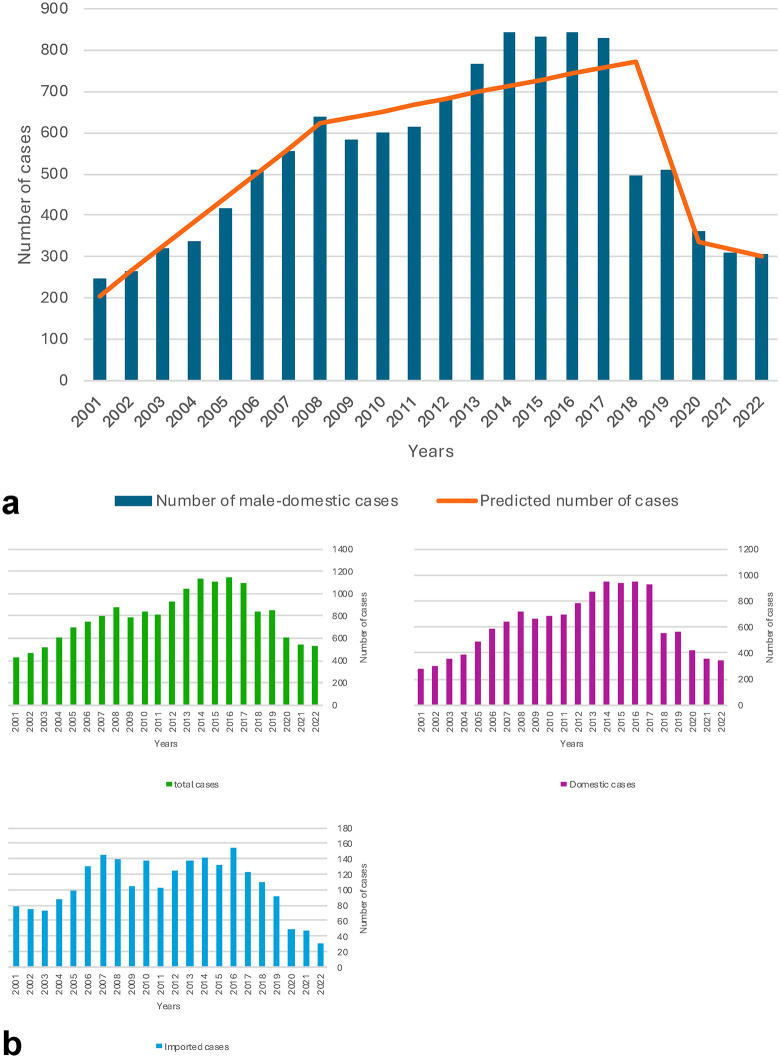
Recent trends of amebiasis in Japan by place of infection. **a**: Male-domestic amebiasis cases reported in Japan from 2001 to 2022 with predicted piecewise time trend. **b**: Amebiasis cases reported in Japan by place of infection (upper-left: total cases, upper-right: domestic cases, and lower: imported cases, 2001-2022).

Estimated slopes from the piecewise regression model for each period (<2008, 2008–2018, 2018–2020, and >2020) are presented in Table 1 with model parameters in S1 Appendix Table B. Before 2008, the number of male-domestic amebiasis cases increased significantly by 59.5 cases per year (p < 0.0001). The trend slowed during 2008–2018, showing a smaller but significant annual increase of 15.2 cases per year (p = 0.0014). A sharp decline occurred during 2018–2020, with cases decreasing by 219.0 cases per year (p < 0.0001). After 2020, the trend did not show statistically significant change (−16.9 cases per year, p = 0.6532). The numbers of male-domestic amebiasis cases reported in Japan from 2001 to 2022 with predicted piecewise time trend is shown in Fig 1a. Bar charts of total, domestic and imported cases from 2001 to 2022 are shown in Fig 1b. Contrast estimates from the Poisson regression model between years 2017 and 2018, and between years 2019 and 2020, for total cases, domestic cases and imported cases are shown in Table 2. Compared with 2017, incidence in 2018 was significantly lower for total and domestic cases, with incidence rate ratio (IRR) of 0.6318 (p < 0.0001) for total cases, and with IRR of 0.5981 (p < 0.0001) for domestic cases. Compared with 2019, incidence in 2020 was significantly lower for total, domestic and imported cases, with IRR of 0.7056 (p < 0.0001) for total cases, IRR of 0.7354 (p < 0.0001) for domestic cases, IRR of 0.5217 (p = 0.0003) for imported cases.

**Table 1:** Slope of prevalence time trend during different periods.

period	time-trend slope	CI		SE	t value	p value
		LL	UL			
**2001-2008**	59.5409	45.58	73.50	7.0176	8.48	<.0001
**2008-2018**	15.1585	6.04	24.28	4.5878	3.3	0.0014
**2018-2020**	−219.04	−276.52	−161.56	28.9126	−7.58	<.0001
**2020-2022**	−16.8753	−91.38	57.63	37.4123	−0.45	0.6532

CI: confidence interval, LL: lower limit, UL: upper limit, SE: standard error.

**Table 2:** Incidence rate ratios in two different time periods (2017-2018 and 2019-2020).

Total amebiasis cases				
Time period	IRR	CI		p value
		LL	UL	
**2017-2018**	0.6318	0.5733	0.6962	<.0001
**2019-2020**	0.7056	0.6266	0.7945	<.0001
**Domestic Amebiasis cases**				
**Time period**	**IRR**	**CI**		**p value**
		**LL**	**UL**	
**2017-2018**	0.5981	0.5384	0.6644	<.0001
**2019-2020**	0.7354	0.6481	0.8346	<.0001
**Imported Amebiasis cases**				
**Time period**	**IRR**	**CI**		**p value**
		**LL**	**UL**	
**2017-2018**	0.8862	0.6848	1.1468	0.3583
**2019-2020**	0.5217	0.3680	0.7396	0.0003

IRR: incidence rate ratio, CI: confidence interval, LL: lower limit, UL: upper limit.

**Fig 2:**
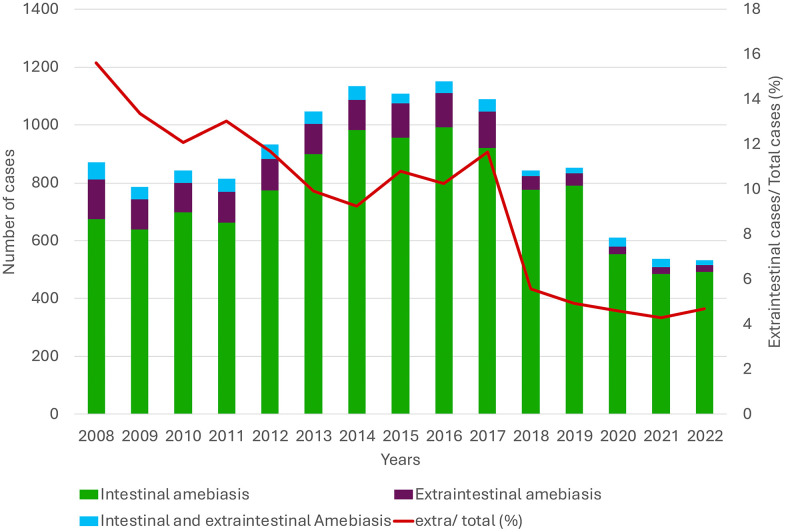
Recent trends of amebiasis in Japan by disease type. Amebiasis cases reported in Japan by disease type (intestinal amebiasis, extraintestinal amebiasis, and intestinal/extraintestinal amebiasis, 2008-2022).

Fig 2 shows the annual trends in amebiasis cases reported in Japan by disease type (intestinal amebiasis, extraintestinal amebiasis, and intestinal/extraintestinal amebiasis) from 2008 to 2022. Through the period, proportion of extraintestinal cases to total cases (line chart in Fig 2) has been declining, which is visually remarkable after 2018.

**Fig 3:**
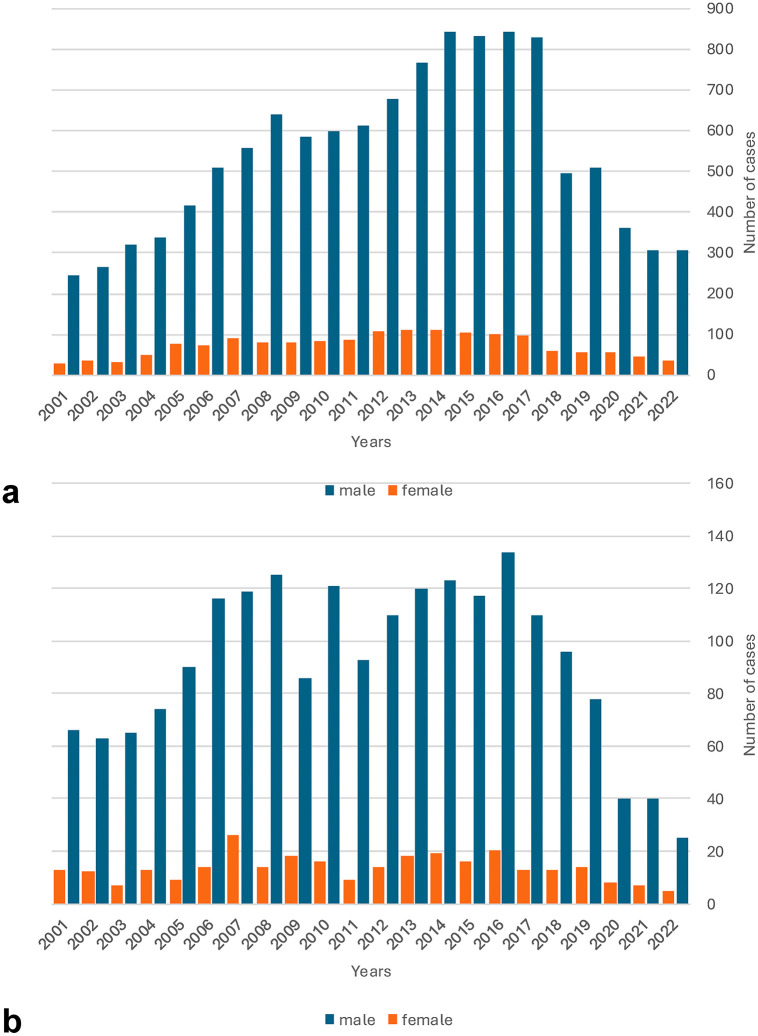
Recent trends of amebiasis in Japan by sex. **a:** Domestic cases of amebiasis reported in Japan by sex (2001-2022). **b:** Imported cases of amebiasis reported in Japan by sex (2001-2022).

The yearly numbers of reported cases of amebiasis in Japan by sex from 2001 to 2022 are shown in Fig 3a (domestic cases) and Fig 3b (imported cases). As previously reported [6], male cases predominated in both domestic and imported cases, and recent years have not seen a change in this trend.

**Fig 4:**
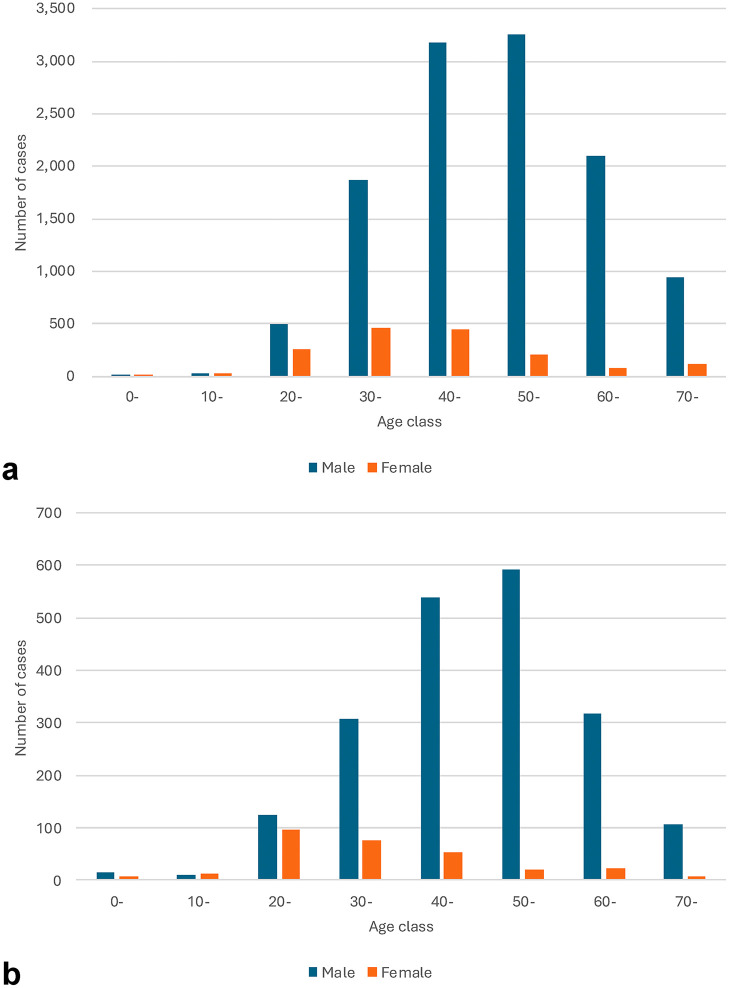
Recent trends of amebiasis in Japan by sex and by age class. **a:** Domestic cases of amebiasis reported in Japan by sex and age class (2001-2022). **b:** Imported cases of amebiasis reported in Japan by sex and age class (2001-2022).

The numbers of reported amebiasis cases in Japan by sex and age class, from 2001 to 2022, for domestic and imported cases are shown in Fig 4a and 4b, respectively. Male cases predominated in the age classes of the 30s to 70s, with the most frequent age classes of male cases being the 40s and 50s.

**Fig 5:**
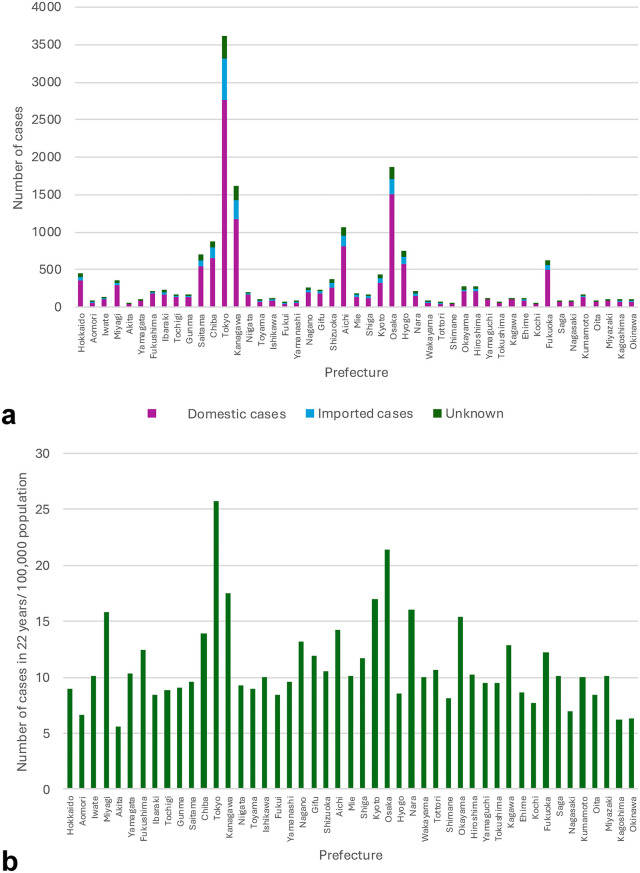
Recent trends of amebiasis in Japan by prefecture. **a**: Amebiasis cases reported in Japan by place of infection and by prefecture (2001-2022). **b**: Amebiasis cases reported in Japan by prefecture (2001-2022)/100,000 population.

Fig 5a shows the number of amebiasis cases by prefecture (2001–2022). As previously reported [6], most amebiasis cases in Japan have been reported in metropolitan areas such as Tokyo, Osaka, Kanagawa, and Aichi prefectures. Fig 5b shows the number of cases per 100,000 population (population data were obtained from estimates by the government [12]. As shown in this figure, there is still a higher incidence in populated areas such as Tokyo and Osaka.

## Discussion

The number of male-domestic amebiasis cases in Japan shows declining trend after 2018 and 2020 (Fig 1a). The number of total and domestic cases decreased significantly in 2018 and 2020 (Table 2) while that of imported cases decreased significantly not in 2018 but in 2020. Overall, it can be stated that declining trend is clearly seen after 2018 and 2020. And decrease in cases numbers in 2020 was observed regardless of place of infection. Hadano et al. showed significant decreases in the numbers of total, intestinal, and extraintestinal weekly reported cases of amebiasis in 2018 and 2019 compared with the preceding years (2014−2017) [8]. In this period, the antibody test reagent stopped being distributed commercially [8,13], which might have reduced the number of reported amebiasis cases. The decrease in case numbers after 2020 coincides with the COVID-19 pandemic, although changes in diagnostic measures after 2018 might have impacted the trend. However, Hirae et al. demonstrated a significant decrease in the total number of amebiasis cases in Japan in the intra-pandemic period (2020−2021) compared with the pre-pandemic period (2015−2019), along with other communicable diseases [9]. Considering this general trend, the present results suggest that the COVID-19 pandemic might have decreased the number of amebiasis cases, regardless of place of infection (domestic or imported). Given that, one can infer that the reduction of amebiasis cases in the intra-pandemic period was brought about not only by the travel ban to and/or from other countries [9], but also by the reduction of socioeconomic activity and/or the increasing awareness of the general public to their sanitation within the country during that period.

Recent epidemiological trends in amebiasis cases in Japan during and after the COVID-19 pandemic period might have behaved differently from other STDs such as syphilis [14] and HIV [15,16]. Reported cases of amebiasis in Japan decreased after 2018 and 2020, and the number has not rebounded up to 2022 (Fig 1a). According to the annual report [5] for 2023, the number of reported amebiasis cases in that year was 489 (the number for 2022 was 533). Reduced numbers of cases in 2020 were also reported for HIV [15] and syphilis [14]. Komori et al. [14] showed a rapid resurgence of the number of syphilis cases reported in Japan in 2021. The rebound trend has also been reported for HIV [16] in 2023. The reason why epidemiological trends differ between amebiasis and these diseases is unclear, and changes in diagnostic measures after 2018 might have affected the amebiasis trend. However, of the three diseases, only amebiasis is in part a disease transmitted by the fecal-oral route, which might have been the key.

Japan is among a few countries where nationwide epidemiological data for amebiasis are available, as well as Taiwan, and trends are similar between the two nations. In Japan, the mode age class of male amebiasis cases was in the 40s and 50s (Fig 4), which is older than that of female cases, with most cases reported in metropolitan areas (Fig 5). In Taiwan, the age class mode of male domestic cases was in the 30s, older than that of female cases [17]. In addition, about 40% of male domestic cases were reported in the capital city, Taipei. For comparison, in Western Sydney, Australia, 88% of confirmed cases of amebiasis were male cases, with a median age of 49 years [18]. Although nationwide epidemiological information is scarce for developed countries other than Japan and Taiwan, we can infer that the same trends in amebiasis cases (male-to-female difference and urban occurrence) might apply to other developed nations.

Inside Japan, there exists epidemiological similarity between amebiasis and HIV infection in terms of the male-to-female ratio and the most frequent age class. Ruzicka et al. analyzed a hospital claims database in Japan, showing that 90% of people living with HIV on antiretrovirals are male, and the most frequent age class was in the 40s [19]. This feature coincides with the epidemiology of amebiasis shown in the present study (Figs 3 and 4), which has been partially mentioned in the previous literature [6,20]. To the best of our knowledge, no reports have shown that amebiasis is spread among MSM using nationwide epidemiological data, but, given the similarity with HIV epidemiology, one can infer that one aspect of the spread of amebiasis is as an STD among MSM.

There are several limitations in this study. First, even though the government has not taken any measures to prevent amebiasis from spreading in recent years, significant reductions of case numbers in 2018 and 2020 might have been due to a natural decrease caused by improvement of general hygiene. Furthermore, considering that there have been several changes in diagnostic measures for amebiasis since 2018, the precise definition of a case of amebiasis can be different in each year; thus, it is difficult to attribute changes after 2020 solely to the COVID-19 pandemic. Second, the NESID reporting system is well established and considered to be reliable, but there may be a discrepancy between the reported numbers and the actual numbers of cases. Of concern, amebiasis cases without subjective symptoms are not diagnosed and not reported. Furthermore, though amebiasis as an STD among MSM was discussed, based on the similarity of its epidemiology with that of HIV, the most frequently reported route of infection [6] is “unknown”; thus, the actual status of amebiasis as an STD remains unclear. Future studies are needed to clarify actual routes of infection of amebiasis, and if possible, describe routes of infection by sex and by age class.

## Conclusion

In conclusion, the number of amebiasis cases reported in Japan showed downward trend after 2018 and 2020. The decrease in 2020 was significant for total, domestic and imported cases, and coincided with the COVID-19 pandemic, which might have reduced the amebiasis case numbers through not only the travel ban to/from overseas, but through less socioeconomic activity during the period. A rebound of case numbers in Japan, which has been seen for syphilis and HIV infection after the COVID-19 pandemic, has not yet been observed for amebiasis. In terms of the male-to-female ratio and the common age classes of reported cases, there is similarity between amebiasis and HIV infection. Why the epidemiological trends in amebiasis behave differently from those of HIV infection and syphilis after the pandemic remains unknown.

## Supporting information

S1 Table Summary tables used for generation of each chart. (XLSX)

S1 Appendix Description of statistical analysis model. (DOCX)
