# Supplementary material for: Epidemiological characteristics of amebiasis in Japan from 2001 to 2022
Source: PLoS One. 2026 Jul 7;21(7):e0318901. doi: 10.1371/journal.pone.0318901 (PMC13340851; doi:10.1371/journal.pone.0318901)
Supplement: S1 Appendix — (DOCX) [file pone.0318901.s002.docx]

**Description of the piece-wise regression model**

$y_{jt} is a number of cases among$group j (male-domestic or other three groups) at time t (2001-2022) assumed to be follow normal distribution with mean $\mu_{jt}$. Mean is modeled as

$$\eta\left( \mu_{jt} \right)=\alpha+\gamma z_{j}+\beta t+\delta z_{j}t+\theta_{1}{z_{j}\left( t-2008 \right)}_{+}+\theta_{2}{z_{j}\left( t-2018 \right)}_{+}+\theta_{3}z_{j}\left( t-2020 \right)_{+}$$

$where \eta is link function, z_{j} is indicator variable assume value 1$ if Male-Domestic and 0 otherwise. $\left( a \right)_{+} is a$ $spline function defined as max\left( 0, a \right)$. First-order autocorrelation was assumed for serial correlation among $y_{jt}$. The above model implies that the rate of prevalence changes at time 2008, 2018 and 2020. The following **Table A** shows slope of regression lines during $t<2008, 2008\leq t<2018, 2018\leq t<2020 and 2020\leq t \mathrm{periods}.$

**Table A**: Model parameters representing slope of regression line

| Period | Slope of regression line |
| --- | --- |
| $t<2008$ | $\beta+\delta z_{j}$ |
| $2008\leq t<2018$ | $\beta+\delta z_{j}+\theta_{1}z_{j}$ |
| $2018\leq t<2020$ | $\beta+\delta z_{j}+\theta_{1}z_{j}+\theta_{2}z_{j}$ |
| $2020\leq t$ | $\beta+\delta z_{j}+\theta_{1}z_{j}+\theta_{2}z_{j}+\theta_{3}z_{j}$ |

$\theta_{1}, \theta_{2}, \theta_{3}$represent rate of prevalence changes at time 2008, 2018 and 2020 respectively. Time trend of male-imported, female-domestic and female-imported group are modeled as $\alpha+\beta t.$

**Results of time trend analysis.**

Estimated model parameters are shown in **Table B**.

**Table B**; Fixed Model Parameter Estimates

| **Parameter** | **Estimate** | **SE** | **df** | **t value** | **p value** |
| --- | --- | --- | --- | --- | --- |
| $\alpha$ | 110.49 | 2173.44 | 81 | 0.05 | 0.9596 |
| $\beta$ | -0.0256 | 1.0805 | 81 | -0.02 | 0.9812 |
| $\gamma$ | -119047 | 14239 | 81 | -8.36 | <.0001 |
| $\delta$ | 59.5665 | 7.1003 | 81 | 8.39 | <.0001 |
| $\theta_{1}$ | -44.3824 | 10.4119 | 81 | -4.26 | <.0001 |
| $\theta_{2}$ | -234.2 | 31.4968 | 81 | -7.44 | <.0001 |
| $\theta_{3}$ | 202.16 | 60.2103 | 81 | 3.36 | 0.0012 |

SE: standard error, df: dgrees of freedom
